# Influence of surgical approach on component positioning in primary total hip arthroplasty

**DOI:** 10.1186/s12891-015-0623-1

**Published:** 2015-08-05

**Authors:** Moritz M. Innmann, Marcus R. Streit, Jeanette Kolb, Jochen Heiland, Dominik Parsch, Peter R. Aldinger, Matthias Königshausen, Tobias Gotterbarm, Christian Merle

**Affiliations:** Department of Orthopaedic and Trauma Surgery, University of Heidelberg, Schlierbacher Landstrasse 200a, 69118 Heidelberg, Germany; Department of Orthopaedic and Trauma Surgery, Karl-Olga-Krankenhaus, Hackstraße 61, 70190 Stuttgart, Germany; Department of Orthopaedic and Trauma Surgery, Paulinenhilfe, Diakonieklinikum, Rosenbergstrasse 38, 70192 Stuttgart, Germany; Chirurgische Universitätsklinik und Poliklinik, Berufsgenossenschaftliches Universitätsklinikum Bergmannsheil, Bürkle-de-la-Camp-Platz 1, 44789 Bochum, Germany

**Keywords:** Minimal invasive approach, Anterolateral approach, Lateral approach, Implant positioning, Hip replacement, Hip arthroplasty

## Abstract

**Background:**

Minimal invasive surgery (MIS) has gained growing popularity in total hip arthroplasty (THA) but concerns exist regarding component malpositioning. The aim of the present study was to evaluate femoral and acetabular component positioning in primary cementless THA comparing a lateral to a MIS anterolateral approach.

**Methods:**

We evaluated 6 week postoperative radiographs of 52 hips with a minimal invasive anterolateral approach compared to 54 hips with a standard lateral approach. All hips had received the same type of implant for primary cementless unilateral THA and had a healthy hip contralaterally.

**Results:**

Hip offset was equally restored comparing both approaches. No influence of the approach was observed with regard to reconstruction of acetabular offset, femoral offset, vertical placement of the center of rotation, stem alignment and leg length discrepancy. However, with the MIS approach, a significantly higher percentage of cups (38.5 %) was malpositioned compared to the standard approach (16.7 %) (p = 0.022).

**Conclusions:**

The MIS anterolateral approach allows for comparable reconstruction of stem position, offset and center of rotation compared to the lateral approach. However, surgeons must be aware of a higher risk of cup malpositioning for inclination and anteversion using the MIS anterolateral approach.

## Background

In the last decade, minimal invasive approaches in primary total hip arthroplasty (THA) have gained growing popularity, providing potential advantages compared to standard approaches such as reduced blood loss, and faster patient recovery [1, 2] as a result of preserved muscle integrity [2]. Reported short-term results have demonstrated good clinical outcomes for minimal invasive approaches (MIS) comparable to standard approaches [3, 4]. However, concerns exist regarding limited surgical exposure of the hip, potentially compromising component fixation and positioning which may have adverse effects on prosthesis longevity [5]. Unequivocal radiological data have been reported for cup inclination and anteversion, identifying minimal invasive approaches as a potential risk factor for cup malpositioning [6–9]. Considering the reconstruction of leg length and femoral offset, comparable results have been reported for minimal invasive and standard approaches [6, 7]. However these studies have only compared the mini incision posterior to a standard posterior or posterolateral approach, without reporting on results for femoral and acetabular offset reconstruction separately [6, 7]. To our best knowledge, there are no studies available focusing on the aspect of cup positioning and concomitant reconstruction of offset and leg length using a minimal invasive anterolateral approach.

Therefore, the aim of the present study was to evaluate femoral and acetabular component positioning in primary cementless THA comparing a lateral to a MIS anterolateral approach.

## Methods

### Study cohort

The present retrospective radiological comparative study included 106 patients, who had undergone 106 consecutive unilateral primary THAs with the same cementless implant components at our institution between January 2004 and December 2007. Patients were followed prospectively with our institutional database and were retrospectively identified for inclusion into the study cohort. Dependent on the surgical approach, patients were assigned either to group A (minimal invasive anterolateral approach [2]) or group B (standard lateral transgluteal approach according to Bauer [10]). Exclusion criteria were defined as bilateral hip disease (Kellgren Lawrence > grade 1) [11], a history of hip surgery prior to THA, previous trauma, metabolic disease and missing pre- or postoperative radiographs. Diagnoses for inclusion were primary osteoarthritis, avascular necrosis of the femoral head or mild dysplasia of the hip (Crowe I) [12]. In total, 52 consecutive patients could be allocated to group A and 54 patients to group B. To evaluate the potential learning curve aspect for cup positioning with the MIS approach, group A was divided into two subgroups. The subgroup A1 comprised the first 26 procedures and subgroup A2 the second 26 procedures. Demographic data is given in Table 1.

**Table 1:** Demographics

Variable	Group A (MIS)	Group B (standard)	P Value
Number of hips	52	54	-
Side (R:L)	30:22	29:25	0.700
Gender (F:M)	32:20	27:27	0.248
Age (years)^a^	64.3 ± 9.9 (35–81)	66.3 ± 12.4 (19–83)	0.783
Body mass index (kg/m^2^) at surgery^a^	25.4 ± 2.6 (18.1-31.0)	26.1 ± 3.7 (18.1-34.2)	0.166
Harris Hip Score at surgery^a^	48 ± 15 (22–90)	54 ± 18 (15–90)	0.088
Devane activity score at surgery^a^	3.1 ± 0.6 (2–4)	2.8 ± 0.6 (2–4)	0.027

^a^Values are expressed as mean ± standard deviation and range in parentheses

Radiographic measurements were performed on pre- and 6 week postoperative low centered anteroposterior (AP) radiographs of the pelvis in both groups. Preoperative body mass index (BMI), Harris Hip score (HHS) [13] and patient activity according to Devane et al. [14] were available for all patients. The study was approved by the institutional review board of the University of Heidelberg (reference 346/2004) and informed consent was obtained from all patients prior to inclusion.

### Surgical procedure and implants

The procedures were performed by 3 senior surgeons in a university hospital setting. The anterolateral approach, according to Bertin and Rottinger [2], was performed with the patient in the lateral position. The standard lateral transgluteal approach, according to Bauer [10], was performed with the patient in the supine position. The standardized peri- and postoperative protocol was identical in both groups, including single-shot antibiotics (Cefuroxime 1,5 g i.v. perioperatively), weight-bearing as tolerated, diclofenac 75 mg daily for the prevention of heterotopic ossification for four weeks and low-molecular weight heparin for six weeks postoperatively as prophylaxis for deep vein thrombosis.

As implants, a cementless tapered titanium straight stem (CLS Spotorno, Zimmer Inc., Warsaw, USA) and a cementless titanium press-fit cup with or without screws (Allofit®/-S, Zimmer Inc., Warsaw, USA) was used in all patients. Femoral implants were available with 3 different neck-shaft angles of 125, 135 and 145 °. In both groups, surgeons aimed for secure press-fit fixation, equal leg length, reconstruction of the preoperative hip offset, neutral stem alignment, cup inclination between 30–50° and cup anteversion between 10–30°. Preoperative planning of the prosthesis size and position was performed on radiographic ap pelvis templates in all cases.

### Radiographic evaluation

Radiographic measurement was performed on digital low-centered AP radiographs of the pelvis [15], by two reviewers (M.M.I., C.M), who were not involved in index surgery. Radiographs were taken with the patient in the supine position and with both legs in 15° internal rotation. The central beam was directed on the symphysis pubis. Correction of magnification of pre-and postoperative radiographs and radiographic measurements were performed according to Dastane et al. [15]. The hip center of rotation (COR) was defined using a circle tool determining the diameter of the femoral head and its center [16]. The femoral offset (FO) was determined as the perpendicular distance between the COR and the proximal femoral shaft axis (FSA) [15, 16]. Acetabular offset (AO) was measured as the perpendicular distance between the COR and line T, with T being the perpendicular line on the transteardrop line (TT) through the ipsilateral teardrop figure [15]. Hip offset (HO) was calculated as the sum of FO and AO [15]. The vertical position of the COR was measured as the perpendicular distance to line TT. Stem alignment was measured as the difference in degrees between anatomic femoral shaft and vertical stem axis [17]. Cup inclination was defined as the angle between the TT line and the line connecting the most superior and inferior aspect of the cup. Cup anteversion was measured and calculated according to the formula by Lewinnek et al. [18], as recently validated by computer tomography based data [19]. Radiographic leg length (LL) was measured as the perpendicular distance between line TT and the apex of the lesser trochanter. Preoperative measurements of all parameters were conducted bilaterally, as all patients had an arthritic and a healthy hip contralaterally before THA. Six weeks postoperatively, radiographic measurements were performed bilaterally again according to the same method. Roman software V1.70 (Institute of Orthopedics, Oswestry, UK) and ImageJ software V1.44 (National Institute of Health, USA) were used for radiographic analysis. Intra- and interobserver reliabilities were calculated for 15 randomly selected data sets of each group, using average-measure intra-class-correlation coefficients (ICC) with a two-way random effects model for absolute agreement. Repeated measurements for intra- observer reliability were performed at day 1 and day 7 in a blinded fashion.

### Statistical analysis

After exploratory data analysis, a Kolmogorov-Smirnov test was performed, testing the variables for normal distribution. As not all variables met the criteria for a normal distribution, non-parametric test were used. Continuous variables between groups were compared using the Mann-Whitney-*U* test and dichotomous variables were compared using a chi-square test. We considered p-values of <0.05 to be statistically significant. Graphpad Prism software V5.01 (Graphpad Software, La Jolla, California) was used to record and analyze the data.

## Results

**Fig. 1:**
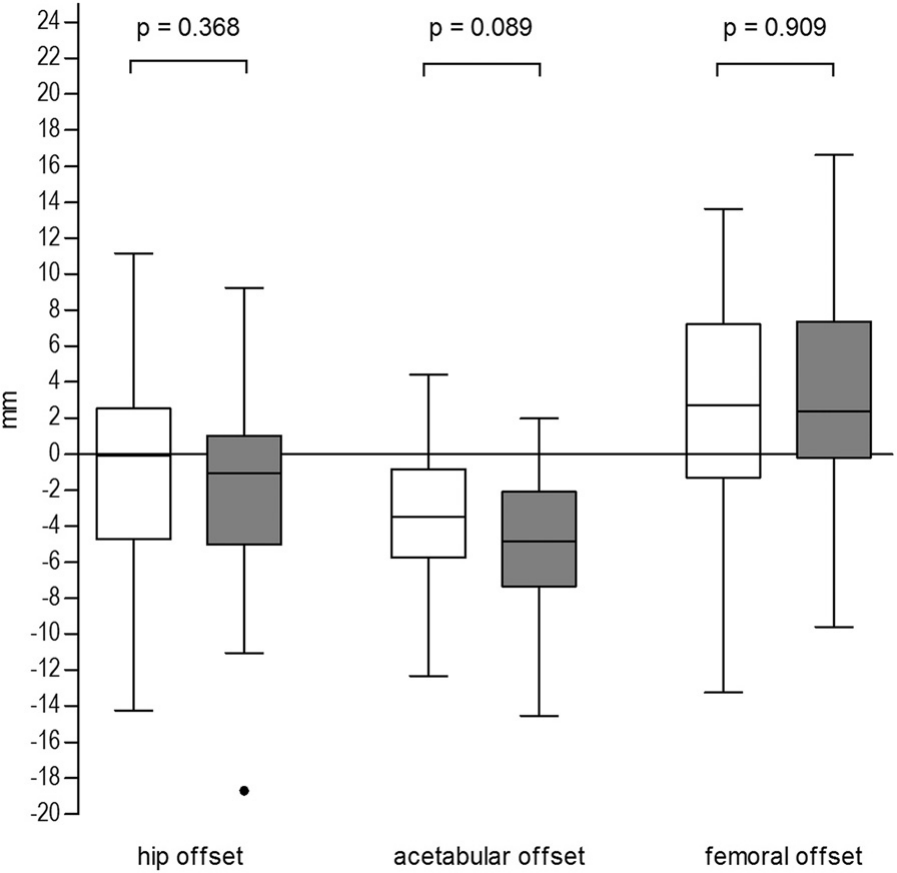
Box plot for postoperative offset difference between healthy and operated hip for MIS anterolateral (white) and standard lateral approach (grey). The box represents the median and interquartile range (IQR), whiskers represent range within 1.5×IQR, dots represent outliers.

**Fig. 2:**
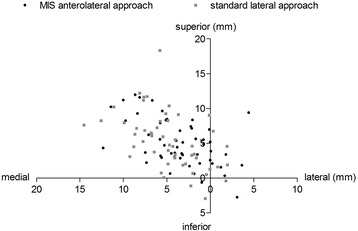
Scatter plot for postoperative position of the COR compared to the contralateral healthy hip for MIS anterolateral (black) and standard lateral approach (grey)

**Fig. 3:**
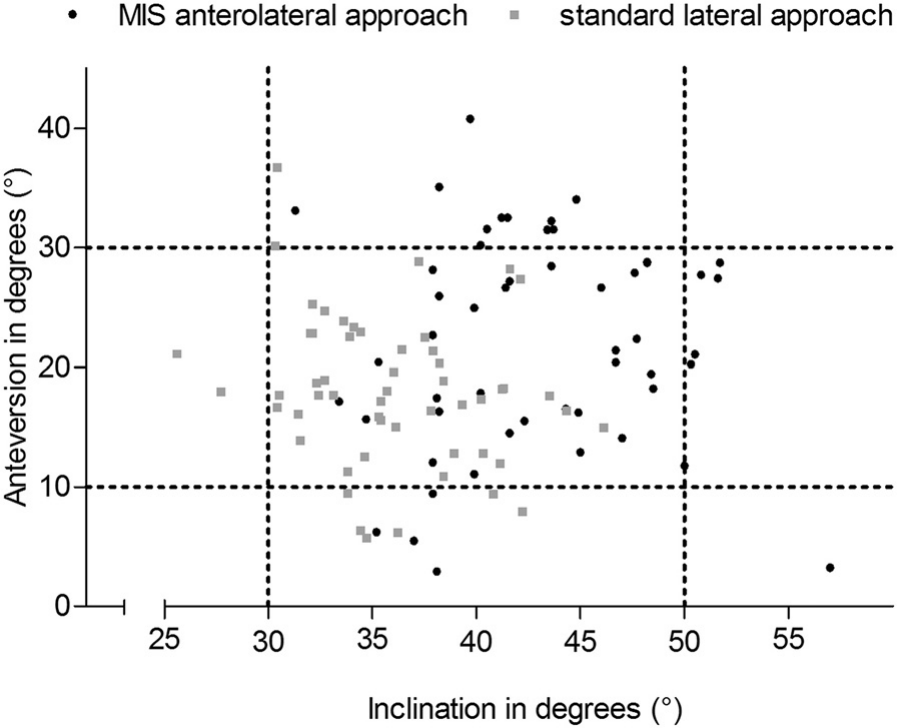
Scatter plot for postoperative cup inclination and anteversion for MIS anterolateral (black) and standard lateral approach (grey)

Intra- and interobserver ICCs for the performed measurements ranged from 0.79 – 0.98. Demographic data and preoperative radiological differences in HO, AO, FO and LL with regard to the contralateral healthy hip were comparable between groups A and B (Tables 1 and 2). Postoperatively, both approaches allowed for accurate reconstruction of HO compared to the healthy contralateral hip (Group A: *p* = 0.697, Group B: *p* = 0.127). In both groups AO decreased, corresponding to a medialization of the COR by 3.5 mm for the MIS (*p* < 0.001) and 4.8 mm for the lateral approach (*p* < 0.001). In contrast, FO increased by 2.7 mm (*p* = 0.027) and 2.9 mm (*p* = 0.010), respectively (Fig. 1). The COR was placed superiorly by 5.1 mm in group A (*p* < 0.001) and 5.3 mm in group B (*p* < 0.001) (Fig. 2). Mean radiographic leg length of the operated limb was increased in both groups without significant difference between both approaches (Group A: 3.0 mm, Group B: 3.5 mm, *p* = 0.354). Mean varus/valgus malalignment of the stem was less than 1° varus and less than 3° valgus in all cases. Stem alignment did not show a significant difference between group A and B (*p* = 0.101). Group A demonstrated a significantly higher percentage of cups outside the target zone for both version and inclination with 38.5 % (anterolateral MIS approach) compared to 16.7 % (lateral approach) (*p* = 0.022) (Fig. 3). In detail 46 (89 %) cups were located in the target zone for cup inclination in group A and 52 (96 %) hips in group B, respectively. For cup anteversion, 37 (71 %) hips were located in the target zone in group A and 46 (85 %) hips in group B, respectively. No learning curve aspect could be detected for cup positioning in our study cohort between subgroup A1 and A2 (*p* = 0.569). Postoperative radiographic measurements are presented in Table 3.

**Table 2:** Preoperative radiographic measurements

Preoperative discrepancy between arthritic and healthy hip	Group A (MIS)	Group B (standard)	*p* Value
Hip offset (mm)	0.6 ± 4.2	0.3 ± 4.1	0.716
Acetabular offset (mm)	2.5 ± 3.2	2.0 ± 2.7	0.444
Femoral offset (mm)	−1.9 ± 4.1	−1.7 ± 3.5	0.892
Leg length difference (mm)	−1.3 ± 4.0	−2.9 ± 5.7	0.088

All values are expressed as mean ± standard deviation

**Table 3:** Postoperative radiographic measurements

Variable	Group A (MIS)		Group B (standard)	
	THA	Healthy hip	THA	Healthy hip
Hip offset (mm)	69.6 ± 7.0	70.5 ± 8.2	69.1 ± 5.6	70.9 ± 7.6
*p* Value	0.697		0.127	
Acetabular offset (mm)	29.4 ± 2.8	32.9 ± 4.0	28.7 ± 3.4	33.4 ± 3.8
*p* Value	<0.001		<0.001	
Femoral offset (mm)	40.2 ± 6.1	37.5 ± 6.0	40.4 ± 4.4	37.5 ± 6.1
*p* Value	0.027		0.010	
Superior placement of the COR (mm)	17.9 ± 3.8	12.8 ± 2.9	18.0 ± 3.4	12.7 ± 3.1
*p* Value	<0.001		<0.001	
Leg length difference^a^ (mm)	3.0 ± 4.8		3.5 ± 4.6	
*p* Value		0.354		
Stem alignment (varus/valgus) (°)	0.8 ± 1.0		0.5 ± 1.0	
*p* Value		0.101		
Cup inclination (°)	42.9 ± 5.4		35.9 ± 4.3	
*p* Value		<0.001		
Cup anteversion (°)	22.0 ± 8.8		18.1 ± 6.2	
*p* Value		0.014		

All values are expressed as mean ± standard deviation; ^a^between healthy and operated hip

## Discussion

The potential advantages of minimally invasive approaches for THA have been attributed to reduced soft-tissue trauma with reported benefits such as reduced blood loss, less postoperative pain, shorter hospitalization time, faster postoperative recovery and better cosmetic results [1, 7, 20]. However concerns have been raised regarding reduced surgical exposure, potentially increasing the risk for neurovascular injury, implant malpositioning, and poor implant fixation [8, 9, 20]. Contradictory results have been reported for cup orientation in dependency of the used surgical approach [4, 8, 9]. To our knowledge, no study is available comparing the MIS anterolateral and the standard lateral approach for offset and leg length reconstruction as well as cup orientation.

The importance of optimal reconstruction of offset and COR is well illustrated in the literature, as it is associated with postoperative abductor strength, range of motion and longevity of the implant [15, 21, 22]. Failures in offset reconstruction may result in complications like limp, pain, fatigue, impingement, dislocation and increased joint reaction forces with higher PE wear [21–26]. Therefore our study sought to evaluate the influence of a MIS anterolateral approach on the reconstruction of the (1) offset, (2) leg length, (3) postoperative position of the center of rotation and (4) cup orientation, compared to a standard lateral approach.

Our finding of comparable restoration hip offset independent of the used approach has not been previously reported. Only Dastane et al. [15] reported a slight postoperative increase of 1.4 mm for hip offset for the mini-posterior approach. In our study, the observed medialization of the COR by 3.5 and 4.8 mm due to acetabular reaming and press-fit fixation could be balanced by a corresponding increase in femoral offset of 2.7 and 2.9 mm, independent of approach. This finding corresponds well to studies of Dorr et al. [7] and Kim et al. [6] reporting an increase in femoral offset of 1.3 to 4.0 mm. Restoration of femoral offset within 5 mm has been associated with a reduction in UHMWPE polyethylene wear, while reduced or enlarged FO of more than 5 mm has been associated with increased PE wear [23, 26]. Adequate restoration of FO and cup position has further been associated with a reduced occurrence of both bony and component femoroacetabular impingement [27, 28]. Therefore, our results for mean femoral offset increase are in the presumably acceptable zone, ranging from 0 to 5 mm for both approaches. The amount of medialization of the COR during acetabular reconstruction has been described before and is consistent with our findings [29, 30]. In the literature, the effect of positioning the COR more medial is controversially discussed. For cemented cups, excessive superior and lateral cup positioning has been demonstrated to have adverse effects [31], as well as superior and medial positioning [32, 33]. However, for uncemented cups no negative effect has been reported for medial positioning of the COR within 5 mm while restoring hip offset [15, 30]. Asayama et al. [21] attributed superior placement of the COR to decreased abductor strength, independent of the used anterolateral or posterior approach. According to these findings, we could demonstrate that the surgical approach did not have an effect on vertical placement of the COR. However, with both approaches the COR was placed superiorly by 5.1 and 5.3 mm in the present cohort. Limited superior placement of the cup may potentially alter the biomechanics of the hip joint. In contrast to excessive (>15 mm) superior cup placement in developmental dysplasia of hip [31], no adverse clinical effects concerning implant survival or postoperative abductor function for limited superior cup placement (≤5 mm) have yet been demonstrated in the literature. Furthermore the present study could demonstrate that leg length reconstruction was independent of the surgical approach. With both approaches, radiographic leg length was increased slightly by 3.0 and 3.5 mm, being within the radiographic discrepancy of less than 6 mm [15].

A significantly higher cup inclination and anteversion could be demonstrated for the MIS anterolateral approach compared to the standard lateral approach. Although mean values were in the target zone of 30–50° for inclination and 10–30° for anteversion [8, 18], the Chi square test demonstrated a significantly higher percentage of cups outside the target zone for the MIS approach. Our finding is supported by the findings of Callanan et al. [8] and Hailer et al. [9], identifying MIS approaches (anterolateral, two-incision, lateral and posterior) as risk factors for cup malpositioning and dislocation in large patient series. Our rate of outliers for cup positioning with the standard lateral approach (12.9 %) compares well to the rate of 12 %, reported for a consecutive series of 1549 hips [34]. In the present cohort, the rate of outliers (38.5 %) with the MIS approach was significantly higher and compared favorably to the rate of 50 % as reported by Malchau et al. [8]. No learning curve aspect could be detected for cup positioning in the present cohort. The last finding should be interpreted carefully, since the number of hips was small in both subgroups, as a result of the strict inclusion criteria. The presented data does further not allow evaluation of surgeon specific learning curves. Interpreting our current results, we believe that accurate cup positioning is still a critical issue in THA. This assumption was recently confirmed by a clinical study of Grammatopoulos et al. [35], indicating that optimal orientation of the cup improves the functional outcome (inclination/anteversion zone of 45°/25° ± 5°). Similar target zones have been identified before in a mathematical models to maximize the range of motion, minimize the risk for cup-neck impingement [36] and minimize wear while maximizing component stability [37]. This small size for the target zone gives reason for concerns, since it cannot be consistently achieved with current technology [35].

There are limitations of the current study that have to be acknowledged. First, due to the retrospective cohort design, patients were not prospectively randomized to a MIS or standard approach, potentially including a selection bias in favor of one approach. However preoperative clinical and radiographic data showed no difference for patients groups, except for a slightly higher activity score in the MIS group. Regarding the radiographic reconstruction of the hip, this potential bias is of limited relevance. Secondly, because of the comparative radiological study design, no postoperative clinical or implant survival data have been obtained, and thus the present study cannot provide any data neither on potential clinical benefits based on reduced soft tissue trauma nor on the influence of component positioning on implant survival. Thirdly, we acknowledge the potential disadvantage of malpositioning the patient in the x-ray beam with consecutive malrotation of the pelvis and femur, potentially limiting the accuracy of the reported radiographic measurements [16]. We tried to minimize this effect by using standardized radiographic techniques and the contralateral healthy hip as control. Hence, the study does not intend to emphasize absolute measurement values. It rather tries to relate the changes in offset and leg length compared to the healthy contralateral hip. Fourthly, we could not provide any measurements on stem anteversion. Therefore we could not address the aspect of combined anteversion technique for cup and stem placement [38]. We believe this technique is an essential part of component positioning and has to be addressed in further clinical studies. The strength of the study results from the comparability of patient collectives in both groups, regarding preoperative demographic and radiographic data. Another strength of the present study is represented by the good reproducibility of the measured parameters as illustrated by intra- and inter observer ICCs.

## Conclusion

Our study demonstrates comparable radiographic reconstruction for the anterolateral MIS and standard lateral approach, regarding hip offset, placement of the COR, stem alignment and leg length. However, the MIS approach was associated with a significantly higher percentage of hips outside the target zone for both cup inclination and anteversion. Therefore, surgeons must be aware of a higher risk of cup malpositioning using the MIS anterolateral approach. Limited medialization of the COR is inevitable to provide sufficient cup press fit and a concomitant increase in FO is necessary to fully restore hip offset. Further studies are needed to evaluate beneficial effects of MIS surgical approaches and concomitant component positioning on postoperative function, component survival, and PE wear.

## Abbreviations

MIS: Minimal invasive surgery
THA: Total hip arthroplasty
AP: Anteroposterior
BMI: Body mass index
HSS: Harris Hip score
COR: Center of rotation
FO: Femoral offset
FSA: Femoral shaft axis
AO: Acetabular offset
HO: Hip offset
TT: Transteardrop line
LL: Radiographic leg length
ICC: Intra-class-correlation coefficients

## References

[1] Berger RA (2003). Total hip arthroplasty using the minimally invasive two-incision approach. Clin Orthop Relat Res.

[2] Bertin KC, Rottinger H (2004). Anterolateral mini-incision hip replacement surgery: a modified Watson-Jones approach. Clin Orthop Relat Res.

[3] Sendtner E, Borowiak K, Schuster T, Woerner M, Grifka J, Renkawitz T (2011). Tackling the learning curve: comparison between the anterior, minimally invasive (Micro-hip(R)) and the lateral, transgluteal (Bauer) approach for primary total hip replacement. Arch Orthop Trauma Surg.

[4] Cheng T, Feng JG, Liu T, Zhang XL (2009). Minimally invasive total hip arthroplasty: a systematic review. Int Orthop.

[5] Floren M, Lester DK (2006). Durability of implant fixation after less-invasive total hip arthroplasty. J Arthroplasty.

[6] Kim YH (2006). Comparison of primary total hip arthroplasties performed with a minimally invasive technique or a standard technique: a prospective and randomized study. J Arthroplasty.

[7] Dorr LD, Maheshwari AV, Long WT, Wan Z, Sirianni LE (2007). Early pain relief and function after posterior minimally invasive and conventional total hip arthroplasty. A prospective, randomized, blinded study. J Bone Joint Surg (Am Vol).

[8] Callanan MC, Jarrett B, Bragdon CR, Zurakowski D, Rubash HE, Freiberg AA, Malchau H (2011). The John Charnley Award: risk factors for cup malpositioning: quality improvement through a joint registry at a tertiary hospital. Clin Orthop Relat Res.

[9] Hailer NP, Weiss RJ, Stark A, Karrholm J (2012). The risk of revision due to dislocation after total hip arthroplasty depends on surgical approach, femoral head size, sex, and primary diagnosis. An analysis of 78,098 operations in the Swedish Hip Arthroplasty Register. Acta Orthop.

[10] Bauer R, Russe W (1984). The transgluteal approach in hip joint arthroplasty. Z Orthop Grenzgeb.

[11] Kellgren JH, Lawrence JS (1957). Radiological assessment of osteo-arthrosis. Ann Rheum Dis.

[12] Crowe JF, Mani VJ, Ranawat CS (1979). Total hip replacement in congenital dislocation and dysplasia of the hip. J Bone Joint Surg (Am Vol).

[13] Harris WH (1969). Traumatic arthritis of the hip after dislocation and acetabular fractures: treatment by mold arthroplasty. An end-result study using a new method of result evaluation. J Bone Joint Surg (Am Vol).

[14] Devane PA, Robinson EJ, Bourne RB, Rorabeck CH, Nayak NN, Horne JG (1997). Measurement of polyethylene wear in acetabular components inserted with and without cement. A randomized trial. J Bone Joint Surg (Am Vol).

[15] Dastane M, Dorr LD, Tarwala R, Wan Z (2011). Hip offset in total hip arthroplasty: quantitative measurement with navigation. Clin Orthop Relat Res.

[16] Merle C, Waldstein W, Pegg E, Streit MR, Gotterbarm T, Aldinger PR, Murray DW, Gill HS (2012). Femoral offset is underestimated on anteroposterior radiographs of the pelvis but accurately assessed on anteroposterior radiographs of the hip. J Bone Joint Surg Br Vol.

[17] Aldinger PR, Jung AW, Breusch SJ, Ewerbeck V, Parsch D (2009). Survival of the cementless Spotorno stem in the second decade. Clin Orthop Relat Res.

[18] Lewinnek GE, Lewis JL, Tarr R, Compere CL, Zimmerman JR (1978). Dislocations after total hip-replacement arthroplasties. J Bone Joint Surg (Am Vol).

[19] Lu M, Zhou YX, Du H, Zhang J, Liu J (2013). Reliability and validity of measuring acetabular component orientation by plain anteroposterior radiographs. Clin Orthop Relat Res.

[20] Berry DJ, Berger RA, Callaghan JJ, Dorr LD, Duwelius PJ, Hartzband MA, Lieberman JR, Mears DC (2003). Minimally invasive total hip arthroplasty. Development, early results, and a critical analysis. Presented at the annual meeting of the American orthopaedic association, Charleston, south Carolina, USA, June 14. J Bone Joint Surg (Am Vol).

[21] Asayama I, Chamnongkich S, Simpson KJ, Kinsey TL, Mahoney OM (2005). Reconstructed hip joint position and abductor muscle strength after total hip arthroplasty. J Arthroplasty.

[22] Cassidy KA, Noticewala MS, Macaulay W, Lee JH, Geller JA (2012). Effect of femoral offset on pain and function after total hip arthroplasty. J Arthroplasty.

[23] Little NJ, Busch CA, Gallagher JA, Rorabeck CH, Bourne RB (2009). Acetabular polyethylene wear and acetabular inclination and femoral offset. Clin Orthop Relat Res.

[24] Barrack RL (1998). Factors influencing polyethylene wear in total joint arthroplasty. Orthopedics.

[25] Devane PA, Horne JG (1999). Assessment of polyethylene wear in total hip replacement. Clin Orthop Relat Res.

[26] Sakalkale DP, Sharkey PF, Eng K, Hozack WJ, Rothman RH (2001). Effect of femoral component offset on polyethylene wear in total hip arthroplasty. Clin Orthop Relat Res.

[27] Malik A, Maheshwari A, Dorr LD (2007). Impingement with total hip replacement. J Bone Joint Surg (Am Vol).

[28] Widmer KH, Majewski M (2005). The impact of the CCD-angle on range of motion and cup positioning in total hip arthroplasty. Clin Biomech.

[29] Bonnin MP, Archbold PH, Basiglini L, Fessy MH, Beverland DE (2012). Do we medialise the hip centre of rotation in total hip arthroplasty? Influence of acetabular offset and surgical technique. Hip International : J Clin Exp Res Hip PatholTherapy.

[30] Dolhain P, Tsigaras H, Bourne RB, Rorabeck CH, Mac Donald S, Mc Calden R (2002). The effectiveness of dual offset stems in restoring offset during total hip replacement. Acta Orthop Belg.

[31] Pagnano W, Hanssen AD, Lewallen DG, Shaughnessy WJ (1996). The effect of superior placement of the acetabular component on the rate of loosening after total hip arthroplasty. J Bone Joint Surg (Am Vol).

[32] Ranawat CS, Dorr LD, Inglis AE (1980). Total hip arthroplasty in protrusio acetabuli of rheumatoid arthritis. J Bone Joint Surg (Am Vol).

[33] Karachalios T, Hartofilakidis G, Zacharakis N, Tsekoura M (1993). A 12–18year radiographic follow-up study of Charnley low-friction arthroplasty. The role of the center of rotation. Clin Orthop Relat Res.

[34] Barrack RL, Krempec JA, Clohisy JC, McDonald DJ, Ricci WM, Ruh EL, Nunley RM (2013). Accuracy of acetabular component position in hip arthroplasty. J Bone Joint Surg (Am Vol).

[35] Grammatopoulos G, Thomas GE, Pandit H, Beard DJ, Gill HS, Murray DW (2015). The effect of orientation of the acetabular component on outcome following total hip arthroplasty with small diameter hard-on-soft bearings. Bone Joint J.

[36] Widmer KH, Zurfluh B (2004). Compliant positioning of total hip components for optimal range of motion. J Orthop Res: Off Pub Orthop Res Soc.

[37] Elkins JM, Callaghan JJ, Brown TD (2015). The 2014 Frank Stinchfield Award: The ‘landing zone’ for wear and stability in total hip arthroplasty is smaller than we thought: a computational analysis. Clin Orthop Relat Res.

[38] Dorr LD, Malik A, Dastane M, Wan Z (2009). Combined anteversion technique for total hip arthroplasty. Clin Orthop Relat Res.

